# Benefits of High-Intensity Intensive Care Unit Physician Staffing under the Affordable Care Act

**DOI:** 10.1155/2011/170814

**Published:** 2011-11-01

**Authors:** Sachin Logani, Adam Green, James Gasperino

**Affiliations:** Section of Critical Care Medicine, Department of Medicine, Drexel University College of Medicine, Philadelphia, PA 19102, USA

## Abstract

The Affordable Care Act signed into law by President Obama, with its value-based purchasing program, is designed to link payment to quality processes and outcomes. Treatment of critically ill patients represents nearly 1% of the gross domestic product and 25% of a typical hospital budget. Data suggest that high-intensity staffing patterns in the intensive care unit (ICU) are associated with cost savings and improved outcomes. We evaluate the literature investigating the cost-effectiveness and clinical outcomes of high-intensity ICU physician staffing as recommended by The Leapfrog Group (a consortium of companies that purchase health care for their employees) and identify ways to overcome barriers to nationwide implementation of these standards. Hospitals that have implemented the Leapfrog initiative have demonstrated reductions in mortality and length of stay and increased cost savings. High-intensity staffing models appear to be an immediate cost-effective way for hospitals to meet the challenges of health care reform.

## 1. Introduction

The Affordable Care Act enacted by the Obama administration has challenged both hospital and physician providers to deliver quality care at low cost. As a result, health care systems must rethink how to deliver care more efficiently while accommodating the needs of a growing population of elderly and newly insured patients. Novel methods of providing health care are needed to provide high-quality care in the face of reduced rates of reimbursement from Medicare and other third-party payers.

New health care delivery models are emerging. One model, the accountable care organization (ACO), relies on an organized multidisciplinary team of physicians and health care providers employed across one or more hospitals to provide cost-effective health care to a defined population [1]. The ACO is reimbursed by Medicare to address all the health care needs of the elderly or disabled. By coordinating care on both a system and a provider level, the ACO truly becomes “accountable” for delivery of health care throughout the patient's clinical course [1]. This model is believed to be a more efficient way to deliver health care because it capitalizes on the benefits and expertise of a multidisciplinary team to reduce fragmentation of care and to improve quality.

The concept of an organized multidisciplinary team accountable for patient care is not new to the field of critical care medicine. In fact, critical care medicine appears to have had a 10-year head start on meeting the challenges of health care reform. However, it was the private sector rather than the federal government that championed these efforts. Perhaps the most influential force from the private sector is The Leapfrog Group (LFG), which entered the health care environment in 2000 (http://www.leapfroggroup.org/). The group is a consortium representing 130 employers and 65 Fortune 500 companies that purchase health care for their employees. The LFG initially focused on improving four areas central to patient safety and cost containment in health care: computerized physician order entry; evidence-based hospital referral; LFG safe practices scores; and intensive care unit (ICU) physician staffing (IPS). 

Arguably, the cost-effectiveness of implementing the LFG standard or other high-intensity IPS models in a hospital critical care system may prove to be the greatest ally for hospitals needing to meet the challenge of health care reform. This observation is especially true for academic medical centers where the recent Accreditation Council for Graduate Medical Education (ACGME)-imposed reductions to resident work hours may further weaken ICU staffing, requiring workflow restructuring to include greater reliance on midlevel practitioners and attending physicians [2, 3]. In addition, the federal government has created multiple platforms to increase transparency of health care outcomes and has imposed quality-based reimbursements on providers.

Hospitals that are compliant with the Leapfrog standard for IPS staff their ICUs with intensivists who are present during daytime hours and provide care exclusively for ICU patients (e.g., the Leapfrog Group standard). Additionally, when not present in house or via telemedicine, the intensivist must respond to pages and arrange for a physician or physician's extender who is certified in the fundamentals of critical care support to reach the ICU within 5 minutes if needed (http://www.leapfroggroup.org/). This staffing model has been shown to decrease mortality rates, shorten ICU length of stay (LOS), and increase cost-effectiveness [4]. Studies suggest that it has the potential to save 53,850 lives per year in the United States [5]. However, wide variability exists in IPS models in US hospitals: only 4% of ICUs met LFG standards for physician staffing in 2000 [6]. In this paper, we evaluate the cost-effectiveness and clinical outcomes associated with implementing the LGF's standard for IPS (e.g., high-intensity IPS) and discuss how this model supports the vision of health care reform. We also identify barriers to nationwide implementation of the LFG IPS standard and offer ways to overcome them. Although the literature uses different terms to describe ICU physician staffing models, in this paper we use “high-intensity IPS” and the “Leapfrog standard for IPS” interchangeably to avoid confusion. 

## 2. Health Care Reform

The Affordable Care Act recently signed into law by President Barack Obama seeks to enhance patient safety and create a high-value, cost-effective health care system. Accomplishing this goal requires focusing on improving the mechanisms by which health care is delivered. The aim of the Affordable Care Act, specifically that of Title III, Improving the Quality and Efficiency of Health Care, is to incentivize institutions and physicians to deliver the highest level of care (http://www.whitehouse.gov/health-care-meeting/proposal /titleiii). Although beds in the ICU account for approximately 10% of total hospital beds, the cost of delivering care to ICU patients is disproportionately high [7]. The cost of providing ICU care ranges from $2000 to $3000 per day at many US hospitals [8], and Medicare beneficiaries currently account for 42% to 52% of ICU admissions [8, 9]. Forty percent of Medicare beneficiaries are admitted to an ICU during their terminal illness, accounting for a quarter of all Medicare expenditures [10, 11]. Strategic use of resources, without compromising the quality of care, is necessary to deliver care to approximately 32 million Americans who were uninsured previously and who are now projected to enter the health care system because of the recent health care reform.

Title III of the Affordable Care Act seeks to improve the quality and efficiency of medical care by placing emphasis on value-based purchasing (VBP). This concept was formally introduced nearly 8 years ago but has evolved considerably in recent years. In 2003, a VBP program called the Hospital Quality Alliance (http://www.hospitalqualityalliance.org/) was initiated through the joint efforts of the public and private sectors [12]. One aim of this program was to create transparency in clinical outcomes for the general public. In 2005, the Centers for Medicare & Medicaid Services launched a Web site for hospital comparisons that provides quality-of-care information to the public. Next, the “pay-for-reporting” initiative of 2007 penalized hospitals that did not submit quality data [13]. The success of the Hospital Quality Incentive Demonstration pay-for-performance project of 2003 serves as the model for the current VBP reform [12, 13].

Value-based purchasing ties Medicare reimbursement to outcome measures by offering incentives to hospitals that meet certain performance standards; thus, it creates a link between outcomes and payment for services. Moreover, VBP holds hospitals accountable for following clinical guidelines and obtaining successful clinical outcomes. Health care providers are encouraged to strive for quality and cost-effective treatment, and reimbursement is based less on quantity of care and more on quality of care. In addition to clinical measures, patient satisfaction is measured using the Hospital Consumer Assessment of Healthcare Providers and Systems survey [14]. Certain high-cost conditions, for example, acute myocardial infarction, heart failure, pneumonia, surgeries, and any health-care associated infection, have been targeted as focus areas for the 2013 fiscal year. Through the use of the Physician Quality Reporting Initiative, other conditions will be identified and appropriate incentives will be provided for quality care. Performance standards will be established on the basis of practical experience, historical performance standards, and realistic improvement rates. This system will facilitate the evaluation of a hospital's total performance. A hospital's performance score will be publicized, adding more incentive to focus on improvement in the targeted areas along with the financial benefit provided.

The LFG standards for value and cost-effectiveness align with the vision of health care reform. Moreover, the LFG's IPS standard appears to be ideally suited to achieve the goals of care mandated by the Affordable Care Act and its VBP initiatives. The LFG focuses on developing cost-effective health-benefit policies that aim to reduce hospital mortality, improve patient safety, and shorten LOS, the latter being a measure of efficiency. The LFG's IPS standard (e.g., high-intensity staffing) is associated with improvement in both clinical outcomes and cost-effectiveness by various mechanisms, including improved management of ICU resources [15–17]. However, only 26% of ICUs in the United States currently report using a high-intensity model to provide critical care [6].

## 3. Decreased ICU Mortality Rates

The medical literature that has accumulated over the past decade in this area suggests that the LFG's IPS standard is associated with improved clinical outcomes for patients treated in medical and surgical ICUs [17, 18]. Although the exact mechanisms that explain this observation are still largely unknown, recent literature suggests that high-intensity IPS with intensivists and the presence of a multidisciplinary team both play key roles [17]. The intensity of IPS reflects the amount of time spent by an intensivist providing care exclusively to patients in an ICU, and the multidisciplinary team consists of nurses, respiratory care practitioners, and clinical pharmacists [17]. Other factors that may explain the association between high-intensity IPS and improved outcomes include rapid access to critical care by an experienced critical care provider and consistent implementation of protocols to deliver evidence-based critical care [17–20].

A number of studies have shown mortality benefits for patients who are cared for in ICUs staffed with intensivists using a high-intensity model [5, 17]. Young and Birkmeyer [5] performed a meta-analysis of nine studies and found that relative reductions in mortality rates associated with intensivist-model ICUs ranged from 15% to 60%. Pronovost et al. [18] performed a meta-analysis to evaluate the association between ICU physician staffing and patient outcomes. The study included data from 26 observational studies (19 articles and seven published abstracts) that resulted in a total of 14,356 and 13,117 patients cared for in ICUs with high- and low-intensity staffing, respectively. In 14 of the 15 studies included in the analysis, high-intensity IPS was associated with reduced ICU mortality. The pooled estimate of relative risk was 0.61 (95% CI, 0.5–0.75) for high-intensity IPS. Moreover, in studies that accounted for case mix, high-intensity IPS was also associated with a decreased ICU LOS. Uusaro et al. [21] examined 23,134 admissions in 18 ICUs over a 3-year period to determine whether ICU mortality was influenced by weekend admissions to the ICU. The authors applied logistic regression analysis to test the effect of different admission times on ICU mortality after adjusting for disease severity. The mortality rate of patients admitted to the ICU on weekends was 1.2 times higher than that of their counterparts who were admitted on weekdays (adjusted ICU mortality odds ratio, 1.20; 95% CI, 1.01–1.43). The authors speculated that low-intensity IPS contributed to this observation, because all but one ICU included in the study lacked full-time intensivist coverage for the duration of the weekend [21].

The association of high-intensity IPS with clinical outcomes has been studied in patients with a variety of diseases including acute lung injury, cancer, and septic shock [22–29]. Using data collected from a prospective cohort study of patients with acute lung injury, Treggiari et al. [28] examined the association of ICU staffing with hospital mortality in 1075 patients admitted to the ICU. Two IPS models were compared in the study: open and closed. Patients treated in closed ICUs had their care transferred to an intensive care team, which included mandatory consultation by an intensivist, whereas patients treated in open ICUs had their care directed by any attending physician with admitting privileges to an ICU. After adjustment for potential confounders, treatment in a closed ICU was associated with reduced hospital mortality (adjusted odds ratio, 0.68; 95% CI, 0.53–0.89; *P* = 0.004) [28]. This observation has been supported by results from other investigations [23–26]. Hawari et al. [29], using a retrospective cohort design, examined the effect of implementing a high-intensity staffing model on the outcomes of critically ill medical patients in an oncology ICU. The authors evaluated clinical outcomes of adult patients admitted to the ICU before and after implementation of the Leapfrog-compliant IPS model. Although disease severity was similar between the groups, the authors found a significant decrease in mortality rate after implementation of the Leapfrog model for IPS (approximately 12% decrease; *P* = 0.0012) [29]. A similar improvement in mortality rate was observed with the implementation of a high-intensity staffing model in a pediatric ICU [27]. Reynolds et al. [22] evaluated the impact of high-intensity staffing for patients with septic shock in a 16-bed university hospital ICU. Mortality in this population was evaluated over two consecutive 12-month periods. During the first interval, physicians without training in critical care medicine staffed the ICU; during the second interval, those formally trained in critical care staffed the ICU. Although between-group differences in age and severity of illness were similar, the mortality rate was significantly lower in the patient group treated by intensivists (74% compared with 57%; *P* < 0.01) [22]. 

The improved outcomes associated with high-intensity ICU staffing are not limited to medical ICUs but also include neurological and surgical ICUs [30–32]. For example, patients with neurological injury experience better clinical outcomes when treated in ICUs staffed by intensivists using a high-intensity model [32]. Dimick et al. [31] assessed the association between daily rounds by an ICU physician and patient mortality rate after hepatic resection. Data were obtained from the Health Services Cost Review Commission for all adult patients who had a hepatectomy (*n *= 569) in the state of Maryland between 1994 and 1998, and these data were integrated with data on ICU staffing obtained from a questionnaire [31]. In this study, the in-hospital mortality rate was 1.5% in hospitals that had daily rounds by an ICU physician and 7.8% in those that did not (*P* = 0.001). Hospital costs and postoperative complications were also higher when an ICU physician did not perform daily rounds. Similar findings have been reported following esophageal resection [30]. Additionally, Pronovost et al. [15] analyzed hospital discharge data from 46 hospitals with ICUs that provided care to 2987 patients who had undergone abdominal aortic surgery. The authors used a questionnaire to collect data on organizational characteristics of the ICU, including physician staffing, nurse staffing, and other processes of care. In multivariate analysis, not having daily rounds by an ICU physician was independently associated with a threefold increased risk of in-hospital mortality (odds ratio, 3.0; 95% CI, 1.9–4.9) [15]. Nathens et al. [33] used data from a large multicenter prospective cohort study to evaluate the effect of ICU staffing (open compared with intensivist model) on in-hospital trauma-related mortality. ICUs that used an intensivist model (i.e., closed unit) were staffed by an ICU service team led by an intensivist or comanaged with a physician board certified in critical care. In open ICUs, the surgeon assumed postoperative care and continued with clinical responsibilities outside the ICU [33]. After differences in baseline patient characteristics were adjusted for, the presence of an intensivist was associated with a large reduction in the in-hospital mortality rate. The relative risk of death was 0.78 (0.58–1.04) for patients treated in an ICU using an intensivist model [33]. One study that failed to confirm the benefits of intensivists did not include an analysis of the intensity of ICU physician staffing or of the impact of a multidisciplinary team on clinical outcomes [34].

Although several studies have evaluated the impact of high-intensity ICU staffing in medical and surgical ICUs, few studies have investigated its impact in coronary care units (CCUs). In recent years, CCUs have experienced a substantial increase in critically ill patients with noncardiovascular disease, and this change in demographics may influence clinical outcomes [35]. In light of this new trend of chronically critically ill patients being treated in CCUs, it is necessary to explore the impact of high-intensity staffing in CCUs. Furthermore, given the evidence supporting improved outcomes in ICUs led by intensivists, there is a growing need for intensivist-trained cardiologists and for the development of critical care training pathways within current cardiovascular fellowship programs [35].

## 4. Cost-Effectiveness

The financial constraints imposed on hospitals by the health care reform law have created an immediate need for a more efficient system to deliver critical care. Moreover, the growing population of elderly and chronically critically ill patients will increasingly require critical care services throughout their adult lives. Benefits of high-intensity IPS include improved quality indicators through evidenced-based practices and cost savings to hospitals by reducing ICU LOS [36]. 

Intensive care unit LOS is an important variable to consider when measuring the cost-effectiveness of high-intensity IPS, because it is a modifiable factor that has a strong association with both cost and quality of care. Prolonged ICU LOS, for example, is associated with hospital-related complications such as ICU delirium, hospital-acquired infection, and pressure ulcers [22, 30–33, 37]. Similarly, a reduction in ICU LOS can lead to a substantial cost benefit for a hospital, because reimbursement rates for prolonged ICU admissions are typically less than the cost of ICU services. The costs of laboratory tests, procedures, and specialized medications used in the ICU can be significant. Moreover, low-intensity IPS models are associated with misuse of these resources [38]. 

A growing number of reports indicate that implementation of high-intensity IPS is associated with reduced ICU LOS [22, 27, 29–37]. Improved quality of care, reduction in inappropriate admissions, avoidance of ICU-related complications, quicker discharge, and less fragmentation of care all contribute to this observation [22, 27, 29–37]. Hawari et al. [29] demonstrated that implementation of a high-intensity IPS model in the care of critically ill cancer patients was associated with a reduction in ICU LOS and improvement in other quality indicators. In this retrospective cohort study, which was conducted over a 3-year period, the authors measured all-cause mortality rates and LOS in patients admitted to the ICU, before and after implementation of a high-intensity IPS model. Annual all-cause mortality declined by approximately 12%, and the average LOS of patients discharged from the ICU alive was reduced by 38% (4.26 days to 2.63 days). Higgins et al. [36] investigated the factors that contributed to prolonged stay in both an ICU and a hospital. Using data from 34 ICUs at 27 hospitals participating in the Health Impact Project during 1998, the authors found that patients without full-time ICU physician involvement in their care were more likely to have longer ICU stays. Similarly, Dimick et al. [30] reported a 73% increase in LOS (7 days) for patients who underwent esophageal resection in ICUs without intensivist staffing. This finding suggests that IPS is a modifiable factor that reduces ICU LOS. Moreover, Pronovost et al. [18] demonstrated in a meta-analysis a reduction in LOS when a high-intensity staffing model was adopted in 14 of the 17 studies included in their systematic review. 

The financial incentives for hospitals to implement high-intensity IPS models run parallel with the incentives for quality. Birkmeyer et al. [39] estimated in their 2001 analysis that if Leapfrog standards were adopted for ICU care, hospital net savings would range from $800,000 for small hospitals to $3.4 million for large facilities. Similarly, Dimick et al. [30] demonstrated that daily rounds by an ICU physician were associated with decreased LOS, lower hospital costs, and decreased postoperative complications. The authors examined hospital LOS, mortality, and costs of care for 366 patients who had undergone esophageal resection. Data were obtained from 35 hospitals over a 4-year period from 1994 to 1998. After adjusting for different hospital characteristics and case mix, the authors found that the lack of daily rounds by a physician trained in critical care was associated with a 73% increase in hospital LOS and a 61% increase in total hospital cost amounting to $8839 more than the median cost [30]. Furthermore, Pronovost et al. [4] reported that a savings of $510,000 to $3.3 million for a 6- to 18-bed ICU was possible when the IPS standard met the Leapfrog requirement.

## 5. Integrating Palliative Care into Critical Care

The prevalence of patients with a diagnosis of chronic critical illness continues to grow, and despite substantial investment in ICU resources, their clinical outcomes remain poor [40–44]. Recent estimates indicate that the annual cost of caring for patients with chronic critical illness exceeds $20 billion [42]. Owing to the progressive nature of terminal and chronic critical illness, end-of-life care for these patients frequently occurs in the ICU setting [40, 41, 43, 44]. State and federal agencies are now focusing their attention on end-of-life care, because overutilization of hospital and ICU resources during the last 6 months of life has been used as a marker for provider-specific performance (http://www.health.ny.gov/professionals/patients/patient_rights/palliative_care/information_act.htm) [44]. A growing literature suggests that several factors determine Medicare spending during end-of-life care, including patient characteristics, physician practice patterns, and geography [44]. Application of VBP mandates to this population will present great challenges to hospital and physician providers, unless alternative treatment options are consistently made available to patients and their families before they enter the ICU or early in their ICU course. 

Nelson et al. [41] describe two clinical models, consultative and integrative, to introduce palliative care into critical care services. The consultative model employs a team of providers with specialized training in palliative care to advise patients, their families, and other health care professionals on options for end-of-life care. In this approach, patients identified as being at highest risk for poor outcomes are selected for consultation [41]. In contrast, the integrative model incorporates the principles of palliative care into the scope of practice of the multidisciplinary ICU team, and palliative care process measures are applied to patients with extended lengths of stay [41]. 

As palliative care becomes firmly established as yet another discipline of critical care medicine, its benefits to the patient and family will be fully realized. However, even in its early stages of programmatic development, integrating palliative care into critical care has improved clinical outcomes. Curtis et al. [40] performed a before-and-after study to assess whether improving the knowledge of end-of-life care among ICU clinicians was associated with the quality of death and dying as assessed by the patient's family and ICU nurses and with ICU LOS. Although the authors found no significant improvement in family-assessed quality of dying or in family satisfaction with care, nurse-assessed quality of dying improved significantly. In addition, reductions in both ICU and hospital LOS were associated with enhanced knowledge of end-of-life care [40].

## 6. Barriers to Implementation

Nationwide implementation of the Leapfrog initiative for IPS has encountered resistance from many hospitals and physicians. Increased costs associated with recruiting fellowship-trained intensivists have posed a major barrier. Additionally, administrative barriers and the lack of an adequate physician workforce have conspired to limit widespread acceptance of intensivist programs. Kahn et al. [45] surveyed 72 hospitals in the United States to identify barriers to implementing the LFG's IPS standards. Of the 47 hospitals that responded, only 21 ICUs could identify an ICU director. Respondents with ICUs that did not meet the Leapfrog standard for IPS cited the following as reasons: lack of ICU staff organization (e.g., lack of ICU directors), loss of income, and increased cost to hospital administration. Although administrative cost is a major barrier to implementing Leapfrog-compliant IPS models in many hospitals, lifestyle change appears to be an important factor limiting acceptance among individual physicians. For example, the duty hours for intensivists often entail shift work, which raises the concern of burnout among intensivists. Other factors related to intensivist burnout include organizational factors such as the relationship between the physician and his or her director, other colleagues, and nurses. ICU directors must recognize these concerns and strive to improve the work environment by decreasing workload (i.e., reducing patient-to-intensivist ratio) and providing academic opportunities for staff intensivists. Other options that should be explored to reduce physician burnout include increasing the availability of physician extenders in the ICU, creating a primary specialty of intensive care medicine, and creating a certification path in intensive care for emergency medicine physicians [46, 47].

With the advent of the ACGME mandates to restrict resident work hours, the demand for intensivists will continue to rise. High-intensity IPS staffing models require 24/7 coverage schemes, and the staffing for night and weekend coverage typically requires additional full-time equivalents. Moreover, the mandates for quality and patient safety imposed by health care reform will likely compound the current intensivist shortage. Angus et al. [9] projected that by 2020 there will be a 22% shortfall in intensive care specialist hours demanded compared to maximum hours supplied. This shortfall will increase to 35% by 2035. Both the federal government and the private sector must ensure competitive payment for critical care services and provide incentives to attract physicians to specialize in critical care. 

Technological advances may help overcome the barrier imposed by limitations in the intensivist workforce. For many hospitals, telemedicine, which facilitates exchange of audio and visual information electronically between various locations, may be an alternative to increasing intensivist full-time equivalents. A growing body of literature supports the use of this technology to improve the efficiency of delivering critical care. Breslow et al. [48] investigated the utility of telemedicine to improve clinical outcomes in two adult ICUs. A total of 2140 patients between 1999 and 2001 were enrolled in the study, and the telemedicine service was utilized from noon to 7 AM (19 hours) every day for 6 months. Both clinical and economic performance were monitored during this period. The authors found that ICU LOS was shorter with remote ICU care (3.63 days; 95% CI, 3.21–4.04 days) than without (4.35 days; 95% CI, 3.93–4.78 days). The observed decrease in LOS appears to be attributable to lower variable costs and higher hospital revenues. The study also demonstrated a decrease in ICU patient mortality: 9.4% with remote ICU care compared with 12.9% without [48]. A recent study by Lilly et al. [49] also showed that implementation of telemedicine was associated with decreased ICU mortality and LOS. The widespread use of such technologies will require cooperation between the federal government, industry, and the health care professions. 

Health care reform may be the solution to overcoming the financial and cultural barriers to widespread implementation of the Leapfrog standard for IPS. Although the initial investment in a high-intensity IPS model may be significant, the potential to increase revenue and quality is clearly a benefit in the long term. Additional research investigating the long-term fiscal benefits of the high-intensity staffing model may be necessary to motivate hospital administrators to implement change.

## 7. Conclusions

The benefits of high-intensity IPS are clear and are a timely fit, given the recent changes in health care policy. Health care reform has set high standards for patient care, imposed value-based reimbursement, and created transparency in clinical outcomes. Implementation of high-intensity IPS models (e.g., the Leapfrog model) appears to be a cost-effective way to meet these challenges. A growing body of literature supports the cost-effectiveness of high-intensity ICU staffing in both medical and surgical ICUs; however, prospective studies are needed to validate these observations. A large variability remains in ICU staffing models among hospitals in the United States. Cultural and administrative barriers prevent nationwide implementation of high-intensity ICU staffing models. At the provider level, the reluctance to relinquish clinical decision making to other physicians, despite improved health outcomes, is a major barrier to implementing high-intensity IPS models. Overcoming these obstacles will require cooperative efforts between hospital administration and health care professionals. At academic centers, limitations placed on resident work hours will likely lead to greater support for high-intensity IPS models. The relationship between IPS and clinical outcomes requires further study to identify specific patient and system factors that are related to health outcomes. Moreover, the impact of the LFG standard for IPS in CCUs must be explored.
